# RNF6 as an Oncogene and Potential Therapeutic Target—A Review

**DOI:** 10.3390/biotech9040022

**Published:** 2020-11-11

**Authors:** Paweł Zapolnik, Antoni Pyrkosz

**Affiliations:** 1 Students’ Scientific Association of Clinical Genetics, Department of Clinical Genetics, Medical College, University of Rzeszów, Al. Kopisto 2a, 35-959 Rzeszów, Poland; 2 Department of Clinical Genetics, Medical College, University of Rzeszów, Al. Kopisto 2a, 35-959 Rzeszów, Poland; antoni.pyrkosz@gmail.com

**Keywords:** molecular targeted therapy, neoplasms, oncogenes, review, RNF6

## Abstract

The *RNF6* gene encodes Ring Finger Protein 6 (RNF6), which functions as a ubiquitin ligase. Its functions are not entirely known, but research shows that it is involved in human cancer development. Initially, this gene was considered to be a tumor suppressor. Numerous statistical analyses on cell lines and animals indicate, however, that *RNF6* functions as an oncogene, involved in signaling pathways, including SHP1/STAT3, AKT/mTOR, Wnt/β-catenin, or ERα/Bcl-xL. Due to this fact, it has become a potential prognostic factor and therapeutic target. Studies in tumor cells and model organisms using inhibitors such as total saponins from *Paris forrestii* (TSPf), ellagic acid, or microRNA molecules show the effectiveness of inhibiting *RNF6*, and through it, the pathways of tumor cell proliferation. The results of the currently available studies are promising, but the function of RNF6 is not fully understood. More research is needed to assess the role of RNF6 and to check the safety and efficacy of inhibitors.

## 1. Introduction

The *RNF6* encodes Ring Finger Protein 6 (RNF6), an E3 ubiquitin ligase. The gene was first cloned in 1999 by McDonald et al. [1], who defined its locus on chromosome 13q12, a frequent rearrangement site in myeloproliferative neoplasms [2]. RNF6 consists of 685 amino acids; has a coiled-coil domain on the N-terminus; and has a RING-H_2_ finger domain on the C-terminus, which is responsible for a ubiquitin ligase activity [1]. Initially, the RNF6 gene was considered a tumor suppressor gene because of somatic mutations in esophageal squamous cell carcinoma (ESCC) [3]. More recent studies indicate that it is instead an oncogene. RNF6, due to its influence on numerous signaling pathways, is associated with various types of cancer, including prostate cancer, gastric cancer, colorectal cancer, breast cancer, and leukemia [4,5,6,7,8]. There are also reports that the *RNF6* can be considered a prognostic factor in some neoplasms [6,9]. Thanks to its function and involvement in many cancers’ pathogenesis, the *RNF6* may be a molecular target in cancer therapy. Research is currently conducted with various *RNF6* expression inhibitors or compounds that inhibit the activation of RNF6 protein-dependent signaling pathways [10,11,12,13]. There is a large group of proteins with a RING finger domain, but RNF6 has not been fully described [14]. Current studies may provide new therapeutic possibilities. Therefore, it seems essential to know the exact function of this protein and determine all possible ways to influence it.

## 2. RNF6 Functions

Ubiquitination is one of the major protein modifications in eukaryotic cells. It may lead to protein degradation by the proteasome, endocytosis, or kinase activation [4]. The ubiquitin’s different effects depend on the attachment of the protein substrate to specific lysine-containing sites (Lys, K). K48-attached protein chains are degraded by the 26S proteasome. The combination of the protein with K6 ubiquitin protects the substrate against proteolysis [15]. On the other hand, binding to K63 activates mainly non-proteolytic functions [16]. Xu et al. analyzed the RNF6 protein’s role in the androgen receptor (AR) activity in prostate cancer cells. Polyubiquitination of AR in the presence of RNF6 takes place at the K6 or K27 site. This variant leads to atypical polyubiquitination, which does not have to conduce to proteolysis [4]. RNF6 also induces atypical polyubiquitination of the glucocorticoid receptor at the K63 site [17].

The RNF6 protein is involved in different signal transduction pathways in various tissues. Through ubiquitination, it regulates the number of crucial proteins in cells and takes part in carcinogenesis [18,19,20].

Studies in laboratory models revealed another linkage between RNF6 and other critical proteins. Tursun et al. demonstrate in mouse embryos and hippocampal cell cultures that RNF6 leads to polyubiquitination and degradation of LIMK1 kinase, a regulator of cellular actin skeleton. It plays an essential role in the growth of neurons. RNF6 indirectly, through the RNF6/LIMK1 axis, regulates axonal growth cones [21].

## 3. RNF6 as a Tumor Suppressor Gene

Research results that may indicate the suppressive function of the *RNF6* have been published in the medical literature. Hu et al. performed a genomic analysis of the DNA from 11 patients with esophageal squamous cell carcinoma who had a family history of upper digestive tract neoplasms. Fourteen regions of loss of heterozygosity (LOH) have been identified, including chromosome 13q [22]. Li et al. analyzed this region in a group of 56 patients with ESCC. They found an 800 kbp locus on chromosome 13q12.11, where a potential tumor suppressor is likely to be located [23]. New light on this topic was brought by Lo et al.’s research, which analyzed two genes by PCR sequencing: *ATP8A2* and *RNF6* in cells from 24 primary esophageal tumors and 16 cell lines [3]. No mutation in the *ATP8A2* gene was detected, while in *RNF6*, three mutations were found in ESCC cells and 1 in the cell line, which resulted in amino acid changes in the protein: R102K, A242T, G244D, and S623N, respectively. The authors also tested DNA from the blood of three patients in whom the above mutations were detected. The blood DNA alleles were not changed, which points out the somatic nature of the mutations formed during the carcinogenesis [3]. We analyzed the above mutations for significance with the online tool Integrative OncoGenomics (IntOGen) [24]. According to International Cancer Genome Consortium (ICGC), Pan-Cancer Analysis of Whole Genomes (PCAWG), and The Cancer Genome Atlas (TCGA) databases, none of them were under positive selection. The loss of heterozygosity and somatic mutations might designate the suppressor character of the *RNF6*. However, other studies, with greater strength of evidence and performed on multiple tissues, show that it functions as an oncogene.

## 4. RNF6 as an Oncogene

Xu et al. performed one of the first studies that show the possibility of the oncogenic nature of RNF6. A study on human prostate cancer tissue revealed increased expression of the *RNF6* in hormone-refractory prostate cancer cells. It has been shown that RNF6, through ubiquitination, can alter the transcriptional activity of the androgen receptor, possibly by facilitating the attachment of co-activating factors. The authors conducted an in vivo experiment with a xenograft model. Human prostate cancer cells C4-2B and CWR-R1 were administered into the left and right flanks of castrated immunodeficient nude mice. The RNF6 was knocked down by shRNA, and the tumor growth was inhibited [4].

Huang et al. exposed that the expression of the RNF6 gene in gastric cancer tissues is more significantly increased than in an adjacent normal gastric mucosa. Knockdown of *RNF6* in gastric cancer cell lines resulted in significant inhibition of cell growth and decreased expression of cyclin D1 and the anti-apoptotic protein Mcl-1 and sensitizing cells to the cytotoxic effects of doxorubicin. The authors finally showed that knockdown of *RNF6* increases the expression of SHP1 protein, which is a negative regulator of STAT3 and thus influences this critical pathway in carcinogenesis [5]. Xu et al. conducted a study on the ESCC cells. They also demonstrated the influence of the RNF6 on the SHP1/STAT3 pathway. RNF6 activated the STAT3 pathway by modulating ubiquitination and degrading SHP1 in the 26S proteasome [11]. Similar studies were managed by Liang et al. in colorectal cancer. Analogously, *RNF6* expression was increased in colorectal cancer cell lines, and its knockdown led to growth inhibition. As in gastric cancer cells, in this case, downregulating the RNF6 stabilized SHP1, inhibiting the STAT3 pathway, which resulted in cancer cell growth inhibition. Besides, the authors performed an in vivo analysis with nude mice. They injected human colorectal cancer HT-29 and SW1116 cells subcutaneously into the armpit and right flank of 4-week old nude mice. The same as in the cell line study, knockdown of RNF6 prosecutes a reduction of colorectal tumor growth [9]. Another way that RNF6 interferes with colorectal cancer cells is the Wnt/β-catenin pathway. Liu et al. showed that RNF6 leads to ubiquitination and degradation of the transcriptional repressor TLE3, which conduces activation of the Wnt/β-catenin pathway [19]. Additionally, *RNF6* was detected as a mutational driver in colorectal adenocarcinoma according to data from the TCGA database. There are truncating or missense, hotspot mutations, which may point out that this gene could act as a potential oncodriver [24,25].

Studies on hematological neoplasms also prove the critical role of RNF6 in neoplastic transformation. Xu et al. revealed the increased expression of the *RNF6* in leukemic cells against normal blood and bone marrow cells. They found that the transcription factor PBX1, together with the helper protein PREP1, led to the upregulation of RNF6 and probably the axis responsible for the oncogenic nature of RNF6 in leukemic cells. Additionally, the authors made a xenograft study. They inoculated human leukemia K562 cells subcutaneously into the right flanks of nude mice. The knockdown of *RNF6* delayed leukemic tumor growth [8]. The results of studies on acute myeloid leukemia cells by Lu et al. suggest that knockdown of *RNF6* inhibits the PI3K/AKT/mTOR pathway. The RNF6 protein itself activates it, possibly by modulating the phosphorylation of AKT and mTOR proteins [10]. A similar study was performed by the team of Ren et al. on multiple myeloma cells, in which *RNF6* expression was significantly increased concerning normal bone marrow cells. The RNF6 protein stabilized the glucocorticoid receptor, leading to the activation of anti-apoptotic proteins Bcl-xL and Mcl-1, followed by tumor proliferation [17].

The authors who studied breast cancer have reached similar conclusions [7,12]. The expression of the *RNF6* was high in both primary breast cancer cells and cell lines. The RNF6 protein promoted ERα expression and stabilized the ERα/Bcl-xL axis, essential for cancer cells’ survival. Interestingly, Zeng et al., performing a study with the mutant RNF6 protein lacking the RING-H2 domain, proved that it stabilizes this axis in a ubiquitination-independent manner [7].

**Figure 1:**
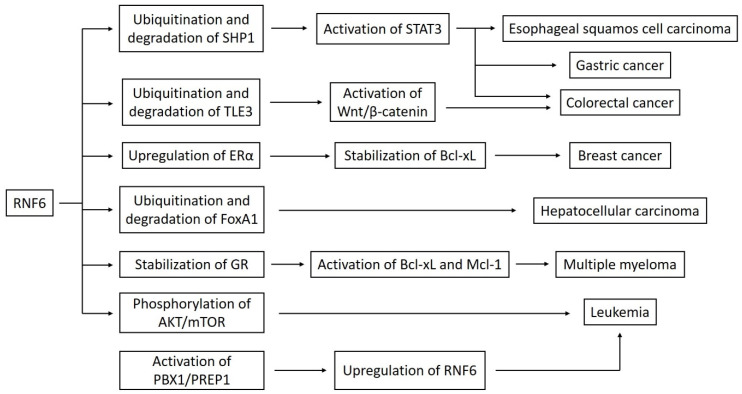
A linkage between RNF6 and various signaling pathways. Abbreviations: ERα–estrogen receptor α; GR–glucocorticoid receptor.

Cai et al. also demonstrated the oncogenic nature of the *RNF6* in hepatocellular carcinoma. The expression of this gene was significantly increased in tumor tissues. RNF6 guided the ubiquitination of a transcriptional repressor FoxA1, which increased radioresistance and epithelial-mesenchymal transition [20]. In their study, Qin et al. made a proteomic analysis of ubiquitination-associated proteins in human lung adenocarcinoma cell line A549. They identified four proteins by MS/MS analysis. One of them was RNF6. The authors compared the proteins’ expression in A549 standard cells and A549 cisplatin-resistant cells, using Western blotting, and observed an increased level of RNF6 in the second group. Treating cells with cisplatin led to the downregulation of RNF6, but the average decrease was lower in the A549 cisplatin-resistant cells. This points out that RNF6 may take part in drug resistance in lung adenocarcinoma [26]. Figure 1 shows a schematic presentation of the connection between RNF6 and different signaling pathways.

Takahashi et al. performed another impressive study. Their team analyzed interactions between p53 and E3 ubiquitin ligases, using a wheat cell-free protein synthesis system (wheat cell-free system) and a high-throughput luminescence-based binding assay (AlphaScreen). They prepared 258 transcriptional templates based on commercial cDNA catalogs. They then synthesized E3 recombinant proteins with the wheat cell-free system. The next step was the assessment of p53 binding, using the AlphaScreen assay. Seven novel E3s, including RNF6, were found to bind with p53. The authors performed an in vitro analysis using H1299 lung carcinoma cells. They revealed that RNF6 bound to p53 and ubiquitinated this protein. The knockdown of RNF6 by small interfering RNAs (siRNAs) led to a significant increase in p53 [27]. The above study may emphasize the role of RNF6 in oncogenesis, in which p53 plays a crucial part.

The above studies indicate a significant role of RNF6 in carcinogenesis. Interference with many signal transduction pathways, receptor stabilization, modulation, or transcription factors’ degradation proves the importance of this protein in cancer biology. To better understand the oncogenic nature of *RNF6* and possible different RNF6 protein interactions at the cellular level, further studies in other cancer cell lines are needed.

## 5. RNF6 as a Prognostic Factor and Therapeutic Target

The finding of new prognostic factors and molecular targets is essential in the development of diagnostics and treatment in modern oncology. Various authors performed the statistical analysis using the Kaplan–Meier method and data from The Cancer Genome Atlas [25]. Zhu and Wang showed that increased RNF6 expression is associated with shorter recurrence-free survival and shorter overall survival in colorectal cancer. It is also interesting that there is a negative correlation between *RNF6* expression and E-cadherin gene expression in colorectal cancer cells, which is related to tumor metastasis. Besides, the evaluation of *RNF6* expression could provide information on the risk of metastasis [6]. Liang et al. presented similar conclusions regarding survival [9]. Liu et al. also confirmed this relationship in their studies, pointing out that increased expression of the *RNF6* is also associated with a higher recurrence risk [19]. Zeng et al. observed the relationship between *RNF6* expression and shorter survival in breast cancer. Moreover, the Chi-square analysis showed that increased expression of *RNF6* is associated with older age, and the upregulation of estrogen and progesterone receptors. Besides, the authors discovered a relationship between the expression of *RNF6* and the migration of neoplastic cells, as well as chemoresistance [7]. Lu et al., in a study on acute myeloid leukemia, presented a similar conclusion: increased *RNF6* expression was associated with shorter survival [10].

One of the most impressive elements of the work devoted to RNF6 is the study of potential inhibitory factors. Lu et al. experimented with saponins isolated from dry roots of *Paris forrestii* (Takht.) H. Li, a plant that has long been used in traditional Chinese medicine [10,28]. Total saponins from *Paris forrestii* (TSPf) inhibited proliferation and induced apoptosis in acute myeloid leukemia cell lines. TSPf induced upregulation of pro-apoptotic proteins, p53, p27, Bax, and Beclin 1, and downregulation of anti-apoptotic proteins, including Bcl-2, Bcl-xL, and Mcl-1. Saponins reduced the expression of *RNF6*, which resulted in inhibition of activation of the AKT/mTOR pathway, crucial in the development of AML. Another element of the study was assessing the use of saponins in a xenograft model in nude mice. K562 human myeloid leukemia cells were injected subcutaneously in female nude mice. TSPf were administered orally at a dose of 100 mg/kg body weight. TSPf significantly reduced the rate of tumor growth without causing a significant decrease in the body weight of mice or abnormalities in laboratory tests, such as blood count, hemoglobin, alanine aminotransferase (ALT), aspartate aminotransferase (AST), or blood urine nitrogen (UN). The authors also showed in the mouse model that TSPf inhibits the RNF6/AKT/mTOR pathway [10].

Another compound with potential anti-cancer properties is ellagic acid, found in pomegranates, black raspberries, and red wine, among others [29,30,31,32]. Xu et al. conducted a study on ESCC cells using ellagic acid. They demonstrate that ellagic acid leads to apoptosis of cancer cells and downregulates anti-apoptotic proteins Bcl-2 and Mcl-1. Ellagic acid inhibits the expression of *RNF6*, which increases the expression of SHP1 and then inhibits the STAT3 pathway [11].

MicroRNAs (miRNAs) are short-chain RNA molecules of approximately 22 nucleotides that do not encode proteins. Their function is to silence protein expression by attaching to appropriate mRNA. Thanks to this, they can participate in various cellular processes, such as metabolism, proliferation, and apoptosis [33,34]. Huang et al. assessed the influence of microRNA-26a-5p (miR-26a-5p) on breast cancer cells. A lower expression of miR-26a-5p occurs in breast cancer and is associated with poorer prognosis. On the other hand, enhancing this molecule’s expression leads to an increase in the concentration of p21, p27, and p53 proteins, regulating the cell cycle and growth inhibition. The authors showed that miR-26a-5p binds to the 3′UTR region of mRNA molecule for RNF6 protein and inhibits its formation, which guides the downregulation of ERα and Bcl-xL [12]. Miao et al. carried out another study using microRNAs. MicroRNA-203a molecule (miR-203a) is downregulated in colorectal cancer cells, whereas its increased expression is conducive to the silencing of RNF6, cell arrest in the G1 phase of the cell cycle, and inhibition of tumor proliferation [13].

Despite limited knowledge of RNF6, the results of the studies mentioned above are promising. RNF6 is involved in the essential pathways of cancer cell proliferation. The use of naturally occurring chemicals or microRNA molecules can yield positive results. A summary of potential anti-RNF6 therapeutic agents is provided in Table 1.

## 6. Challenges and Perspectives

Modern techniques of molecular biology, genetics, and cytophysiology make it possible to assess the function of various genes and their protein products in multiple tissues accurately. Ubiquitin ligases may present different features in different cells because ubiquitination is one of the primary protein modifications. The RNF6, belonging to this family, is not entirely examined. However, more and more studies are performed on primary cancer cells, cell lines, and model organisms, thanks to which we can understand the essential functions of this protein. We currently know that the *RNF6* functions as an oncogene rather than as a tumor suppressor gene. It is associated with many signaling pathways in the cell, which are often involved in carcinogenesis. The performed statistical analyzes and data from cancer genome databases show a significantly increased expression of this protein in neoplastic cells. Therefore, clinicians can use it as a prognostic factor in some cancers, such as colorectal cancer and breast cancer. However, we still do not know how this protein behaves in other tissues and whether it can affect other cancers’ development. More research in this area is needed.

The first studies with the use of RNF6 inhibitors in cell lines and mice showed promising results. It seems appropriate to continue working on the use of natural origin compounds, such as ellagic acid and total saponins from *Paris forrestii*. Positive results from testing with microRNAs also require further development. It is essential to examine the above inhibitors in other tests, on different cell lines, and determine the wide range of safety of these compounds in model organisms. An appropriate follow-up would be the introduction of multicenter, randomized human clinical trials. Thanks to this, we would have the opportunity to introduce another drug directed at a specific molecular target, which could be used in various cancers. Such an agent primarily should be safe and effective, so further research on RNF6 inhibitors is necessary.

## Figures and Tables

**Table 1:** Summary of the potential therapeutic agents against RNF6.

Inhibitor/Potential Therapeutic Agent	Effect	Cancer Type	Inhibited Pathway	Study Model	Reference
Total saponins from *Paris forrestii*–TSPf	Downregulating RNF6 expression, inhibiting proliferation, promoting apoptosis, upregulating pro-apoptotic proteins, downregulating anti-apoptotic proteins	Acute myeloid leukemia	AKT/mTOR	Human myeloid leukemia cell lines K562 and HL-60; K562 cells xenograft in nude mice	Lu et al. [10]
Ellagic acid	Downregulating RNF6 expression, inhibiting proliferation, promoting apoptosis, downregulating anti-apoptotic proteins	Esophageal squamous cell carcinoma (ESCC)	SHP1/STAT3	ESCC cell lines Eca-109 and TE-1	Xu et al. [11]
MicroRNA-26a-5p	Downregulating RNF6 expression, inhibiting cell growth, upregulating cell cycle regulatory proteins	Breast cancer	ERα/Bcl-xL	Breast cancer cell lines MCF-7 and T47D	Huang et al. [12]
MicroRNA-203a	Downregulating RNF6 expression, inhibiting cell growth, arresting cell cycle in the G1 phase	Colorectal cancer	-	Human colon cancer cell lines HCT116 and SW480	Miao et al. [13]
